# The Oxindole Derivatives, New Promising GSK-3β Inhibitors as One of the Potential Treatments for Alzheimer’s Disease—A Molecular Dynamics Approach

**DOI:** 10.3390/biology10040332

**Published:** 2021-04-15

**Authors:** Przemysław Czeleń, Beata Szefler

**Affiliations:** Department of Physical Chemistry, Faculty of Pharmacy, Collegium Medicum, Nicolaus Copernicus University, Kurpińskiego 5, 85-096 Bydgoszcz, Poland; beatas@cm.umk.pl

**Keywords:** GSK-3β, oxindoles, docking, molecular dynamics, inhibition

## Abstract

**Simple Summary:**

Enzymatic overexpression is a determinant of the development of many diseases. Increased activity of the GSK-3β enzyme is a factor that manifests itself in the development of numerous disease entities such as Alzheimer’s disease, schizophrenia, diabetes and cancers. An important medical procedure in such cases is the inhibition of enzyme activity. Based on the comprehensive use of computational chemistry methods, a group of new compounds derived from 2-oxindole was designed. The conducted research allowed the assessment of the conformational properties of the ligand molecules in the GSK-3β active site, the dynamic stability of the obtained complexes and their exact energetic characteristics. Taking into account the obtained data, a narrow group of derivatives showing an affinity for the active site of the GSK-3β enzyme was selected. The comparison of binding properties of selected 2-oxindole derivatives with an inhibitor with confirmed pharmacological activity indicates the high application potential of the newly developed compounds.

**Abstract:**

The glycogen synthase kinase 3β (GSK-3β) is a protein kinase involved in regulating numerous physiological processes such as embryonic development, transcription, insulin action, cell division cycle and multiple neuronal functions. The overexpression of this enzyme is related to many diseases such as schizophrenia, Alzheimer’s disease, diabetes and cancer. One of the basic methods of treatment in these cases is the usage of ATP-competitive inhibitors. A significant group of such compounds are indirubin and its analogs, e.g., oxindole derivatives. The compounds considered in this work are 112 newly designed oxindole derivatives. In the first stage, such molecular properties of considered compounds as toxicity and LogP were estimated. The preliminary analysis of the binding capabilities of considered compounds towards the GSK-3β active site was conducted with the use of the docking procedure. Based on obtained molecular properties and docking simulations, a selected group of complexes that were analyzed in the molecular dynamics stage was nominated. The proposed procedure allowed for the identification of compounds such as Oxind_4_9 and Oxind_13_10, which create stable complexes with GSK-3β enzyme and are characterized by the highest values of binding affinity. The key interactions responsible for stabilization of considered ligand–protein complexes were identified, and their dynamic stability was also determined. Comparative analysis including analyzed compounds and reference molecule 3a, which is also an oxindole derivative with a confirmed inhibitory potential towards GSK3B protein, clearly indicates that the proposed compounds exhibit an analogous binding mechanism, and the obtained binding enthalpy values indicate a slightly higher binding potential than the reference molecule.

## 1. Introduction

Protein kinases are a large group of enzymes involved in the regulation of all important cellular processes. An example of such macromolecules is glycogen synthase kinase-3β (GSK-3β) [1,2,3]. This protein is a well-known representative of serine/threonine protein kinases. The significant interest in this protein is dictated by its important role in the regulation of numerous processes such as embryonic development, transcription, insulin action, cell division cycle, cell differentiation, cell death and multiple neuronal functions. The considerable involvement in the regulation of a variety of processes causes the overexpression of this protein to be associated with the occurrence of many diseases such as schizophrenia [4], Alzheimer’s disease [5,6], bipolar disorder [7], heart hypertrophy [8,9], renal diseases [10], diabetes [11,12] and cancer [12,13]. The primary method of treatment in these cases is the use of ATP-competitive inhibitors exhibiting affinity for the GSK-3β protein. A large group of competitive inhibitors of GSK-3β was created based on indirubin, a substance that has been widely used in traditional Chinese medicine [14,15]. Numerous studies show that indirubin and its derivatives and analogs exhibit significant inhibition potential towards GSK-3β [16,17]. An important group of compounds exhibiting pharmacological potential are oxindole derivatives, which can be classified as indirubin analogs. The inhibition potential of such compounds towards kinases has been a subject of numerous studies [18,19,20]. The common element of all substances belonging to this class of compounds is the oxindole molecule, which exhibits the ability to create stable interactions with amino acids from the GSK-3β active site. Such a molecular core is an ideal starting point in the development of a new group of competitive inhibitors. In a previous study, a procedure was proposed for creating new inhibitors of cyclin-dependent kinase 2 (CDK2) protein based on the oxindole molecule [21].

**Figure 1:**
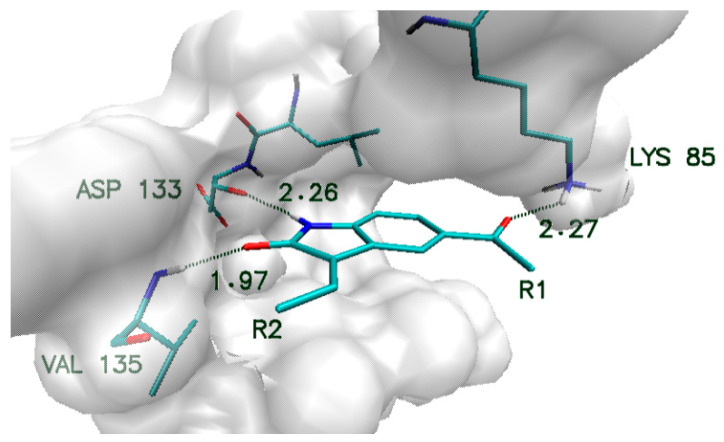
Characteristic interactions of the oxindole core with amino acids from GSK-3β active site.

**Figure 2:**
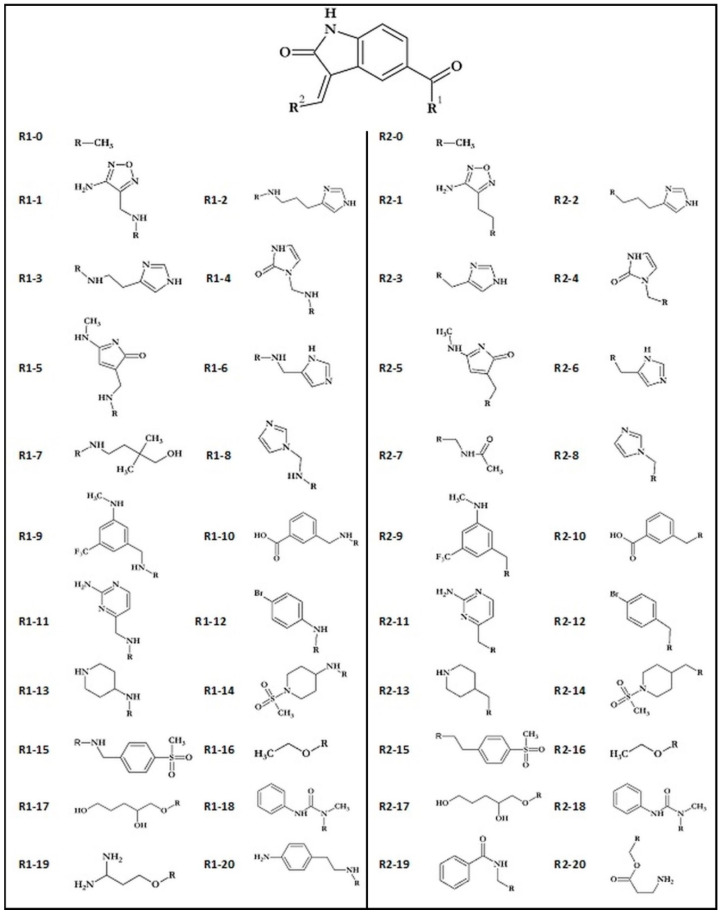
Oxindole core and functional groups used during creation of ligands.

Obtained compounds proved to have significant binding capabilities towards the active site of the CDK2 enzyme. Significant structural similarity between CDK2 and GSK-3β active sites suggests that this class of compounds will have a comparable affinity for both enzymes. The structure of the oxindole core used in the creation of new compounds and its interactions with amino acids from the GSK-3β enzyme are presented in Figure 1. Numerous structural research studies of GSK-3β complexes show that such amino acids as VAL135, ASP133 and LYS 85 play a crucial role in the stabilization of ligands in the area of the enzyme active site. The distribution of hydrogen bond donors and acceptors in the proposed molecular core provides the possibility of creating bonds with all mentioned amino acids. The modification of the native molecule by the addition of substituents to the side chains allows the optimal utilization of the binding capacity of the rest of the amino acids forming the analyzed active site. The set of proposed modifications of the oxindole molecular core is presented in Figure 2. The virtual screening of oxindole derivatives based on the assessment of their binding affinity, as well as their conformational and molecular properties, allows for the design of new inhibitors well suited to the GSK-3β active site.

## 2. Materials and Methods

The first stage of ligand creation relied on a single modification of the molecular core with the use of chosen functional groups supporting varying binding impacts, including hydrogen bond donors and acceptors and hydrophobic interactions. The considered molecular core and all functional groups used are presented in Figure 2. Conducted preliminary docking calculations allowed for selecting substituent groups, the addition of which to particular side chains gives the highest increase in binding affinity towards GSK-3β active site. The use of 10 chosen functional groups in the R1 position and 12 chosen functional groups in the R2 position allowed for the creation of 112 different disubstituted oxindole derivatives.

The names of created derivatives consist of the prefix “Oxind_” and the numbers of the substituents attached to both side chains (R1 and R2). The geometries of ligand structures were optimized with the use of TURBOMOL at COSMO-BP-tzvpd-fine level of theory [22]. The toxicity of considered ligands was estimated using a combination of the 3D/4D QSAR BiS/MC and CoCon algorithms [23,24] with the use of Chemiosophia application [25]. The octanol–water partition coefficient (LogP) was determined with the use of COSMOTHERM application [26].

The docking simulations, including analysis of ligand interactions with protein, were conducted with the use of AutoDockVina [27]. The structure of glycogen synthase kinase 3β (GSK-3β) (PDB ID: 1Q41) [1] was obtained from Brookhaven Protein Database PDB. All molecules used during the docking stage, namely ligands and protein, contain only polar hydrogen atoms. All preliminary procedures were conducted with the use of AutoDock Tools package [28]. In the cases of ligand–GSK-3β protein docking simulations, the dimensions of the grid box were fitted to the size of the enzyme active site (22 × 22 × 16 Å).

The complexes of ligands exhibiting the best binding capabilities towards the GSK-3β active site were used in the next step of modeling, including molecular dynamics simulations. In the case of ligand molecules used in this stage, their structures were characterized with the use of a generalized AMBER force field, while the atomic charges were calculated according to the Merz–Kollmann scheme via the RESP procedure at HF/6-31G* level [29]. The properties of GSK-3β protein were described with the use of ff14SB Force Field [30]. Each ligand–protein complex was neutralized and immersed in a periodic box of TIP3P water box. The considered systems were heated to 300 K by 100 ps of initial MD simulation, while the temperature was controlled by Langevin thermostat [31]. The SHAKE algorithm and periodic boundary conditions were applied for 80 ns of molecular dynamics run. The first 20 ns of molecular dynamics simulation was used as equilibration interval, while the next 60 ns was used in the analysis of ligand–protein active site interactions. Structural analysis was performed with the use of VMD package [32]. The characteristic of energetic aspects of ligand–protein active site interactions was obtained with the use of molecular mechanics Poisson–Boltzmann surface area (MMPBSA) method [33]. In all molecular dynamics simulations, the AMBER 14 package was used [34]. During structural analysis, including the description of interactions occurring in the active site, the hydrogen bonds were defined by the following criteria: distance between donor (D) and acceptor (A) < 3.5 Å, D–H–A angle > 90° and H–A distance < 3 Å.

## 3. Results and Discussion

The basis used to create a new group of competitive inhibitors of the GSK-3β protein was the core created by the oxindole molecule. The proposed structure exhibits significant affinity towards the considered enzyme, which is related to the creation of numerous interactions with crucial amino acids localized in the active site (Figure 1) [1,16]. The increase in binding capabilities of the native molecule was realized by the addition of new substituents as side chains connected to the molecular core. The initial stage was related to a single modification placed in one of two binding sites that were considered during the design of new ligand molecules. Preliminary docking screening was realized for all monosubstituted oxindole derivatives, and the values characterizing obtained complexes are presented in Table 1. The analysis of binding affinity values characterizing all structures enabled the selection of the functional groups that caused the highest increase in binding capabilities of ligand molecules when used. In the case of substitution realized in the R1 position, 17 functional groups increased the binding activity of ligands towards the GSK-3β active site. The observed increase in binding affinity is placed in the range from 1.2 to 20.2%. The confrontation of obtained values with structural properties of functional groups used shows an important relationship. The increase in ligand binding capabilities is strictly related to the presence of aromatic systems, which indicates a significant contribution of hydrophobic interactions in this part of the active site. All substituents for which a decrease in ligand binding properties was noted are aliphatic systems containing hydrogen bond donors and recipients. The substitutions realized in the R2 binding site allowed for the creation of 18 compounds exhibiting better binding capabilities than the native molecule.

The increase in binding affinity noted for that group of oxindole derivatives is in the range from 2.4 to 24.3%. In the case of the second group of substituted monoderivatives, the best effects were also achieved by using substituents with aromatic systems that additionally contain hydrogen bond donors or recipients.

For further work in the design of disubstituted derivatives, substituents that increased the binding capacity of the native molecule by at least 10% when applied were used. In this way, the number of substituents in the R1 position was reduced to 10 and that in the R2 position was reduced to 12. The final group of oxindole derivatives obtained during this procedure consisted of 112 molecules. For each of the 112 ligands, the binding affinity towards the GSK-3β active site, along with other molecular properties such as toxicity and LogP, was estimated during the docking procedure. Table 2 presents values characterizing chosen ligand molecules exhibiting the best properties. The whole set of values characterizing all molecules considered in this work is presented in the Supplementary Materials (Supplementary Materials Table S1). The selection of oxindole derivatives for the next stage of modeling was realized by taking into account many factors. The first was the toxicity of considered molecules. The QSAR algorithm used allows for the prediction of such a molecular property, which is expressed by normalized values in the range from 0 (nontoxic) to 1 (toxic). The method used defines three classes of compounds, namely nontoxic compounds characterized by values in the range from 0 to 0.2, moderately toxic compounds characterized by values in the range from 0.2 to 0.8 and toxic compounds characterized by values over 0.8. The standard error of toxicity value estimation is 0.1. In the analyzed population, 41 obtained compounds can be classified as nontoxic. For the next stage of analysis, the compounds that were characterized by toxicity not exceeding the 0.4 value were selected. This criterion was met by 65 of the considered oxindole derivatives.

The next important criterion was the value of inhibition constant (IC) estimated based on the binding affinity values obtained during the docking stage. The constant was determined based on the relationship presented by the following equation:

$$IC=exp^{\left(\frac{\Delta G_{b}}{RT}\right)}$$

where Δ*G_b_* represents binding affinity, *T* represents temperature and *R* is the gas constant.

**Figure 3:**
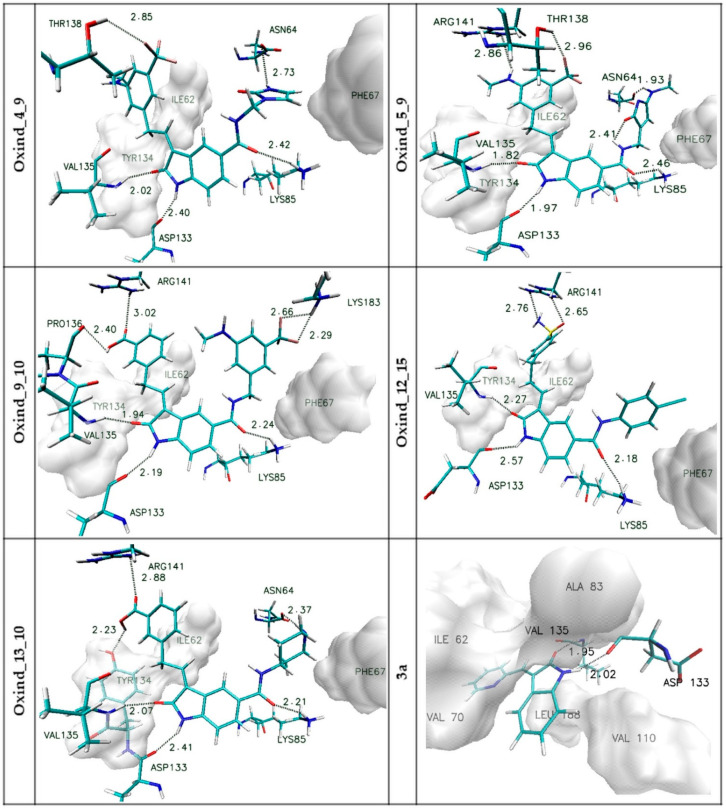
The graphic representations of interactions of oxindole derivatives with the GSK-3β active site identified during the docking stage. The markings on the left side of individual figures are the names of ligands forming complexes with the active site.

All chosen oxindole derivatives exhibit a significant increase in binding capabilities relative to the reference system. In the case of binding affinity, the observed increase is placed in the range from ~30 to ~38%. Observed discrepancies are more noticeable in the context of inhibition constant values, i.e., the reference system, characterized by IC value of 696.17 nM, exhibits 221.6 times worse inhibiting properties towards GSK-3β active site than the best inhibitor obtained (Oxind_13_10). The last factor used during the analysis was the octanol–water partition coefficient (LogP). Based on the LogP values, compounds that could be characterized by low permeability through cell membranes were excluded. Based on the proposed above determinants, the five most promising compounds were selected for the next stage of calculations. It was important to study the structural properties of their protein complexes obtained during the docking stage. The most important interactions involved in the stabilization of complexes created by chosen ligands with the GSK-3β active site are presented in Figure 3; moreover, an additional summary of all identified interactions is provided in Supplementary Materials Table S2.

In all considered oxindole derivatives, three hydrogen bonds created by molecular core atoms with amino acids from the active site, namely ASP133, VAL 135 and LYS 85, are observed. Based on the geometric classification of hydrogen bond strength, the distances noted for such interactions (Supplementary Materials Table S2) allow such impacts to be classified as medium strength hydrogen bonds.

This observation agrees with previous investigations describing the activity of similar compounds towards GSK-3β protein [35,36,37]. The presence of functional groups in both side chains of considered ligands is the source of further interactions stabilizing the considered complexes.

The activity of side chains connected to the R1 position participates in the creation of hydrogen bonds with ASN64 (Oxind_4_9, Oxind_5_9, Oxind_13_10) and LYS183 (Oxind_9_10). In the case of all considered molecules, aromatic systems from this side chain are localized near the hydrophobic part of the active site, the center of which is localized near the PHE 67 residue. The third group of impacts comprises interactions involving chemical groups from side chains connected to the R2 position. In this case, most often observed are hydrogen bonds created with ARG141, which is involved in the stabilization of complexes of all considered ligands; however, such interactions should be classified as weak hydrogen bonds. Additional hydrogen bonds are created with THR138 (Oxind_4_9, Oxind_5_9), PRO136 (Oxind_9_10) and THR134 (Oxind_13_10), and all of them can be classified as medium- or weak-strength interactions. The next important stabilizing factor may be hydrophobic interactions of the hydrophobic pocket created by amino acids such as TYR 134 and ILE 62 with aromatic systems from the ligand side chain.

The complexes of five chosen oxindole derivatives with GSK-3β were the subject of further research realized with molecular dynamics simulations. Obtained outcomes not only allowed broadening the knowledge about the basic interactions stabilizing the considered complexes but also enabled the assessment of dynamic properties of ligands and protein forming the analyzed systems. In order to reliably evaluate the obtained data, analogous simulations were carried out using a reference molecule. 3-(2-Pyridinylmethylene)-2-oxindole (3a) was selected for this task, being a compound containing an analogous oxindole core and a substituent in position 2. The inhibitory abilities of this molecule relative to the GSK-3β protein unequivocally confirm the outcomes presented by Lozinskaya et al. [38]. The first and one of the most primary aspects of the analysis of considered systems is the assessment of structural stability of molecular systems obtained during molecular dynamics simulation. A parameter that can properly describe this property is the root-mean-square deviation (RMSD), which was estimated for all elements of analyzed systems (ligands and protein). Obtained values are presented in two forms, as charts of distributions depending on time (Figure S1) and as average values with corresponding standard deviations (Table 3). In the case of the protein part of considered complexes, all presented distributions have very similar properties, such as duration of structural equilibration period, comprising approximately the first 20 ns of simulation time, and maximal values and their fluctuations, with average values placed in the range from 2.28 to 2.61 and adequate standard deviations (from 0.17 to 0.21). The values describing the behavior of individual ligand molecules are, on the other hand, much more diverse. Among the analyzed ligands, similar molecules characterized by significant structural rigidity and limited mobility in the space of the active site, as well as systems characterized by significant structural flexibility, can be distinguished.

**Figure 4:**
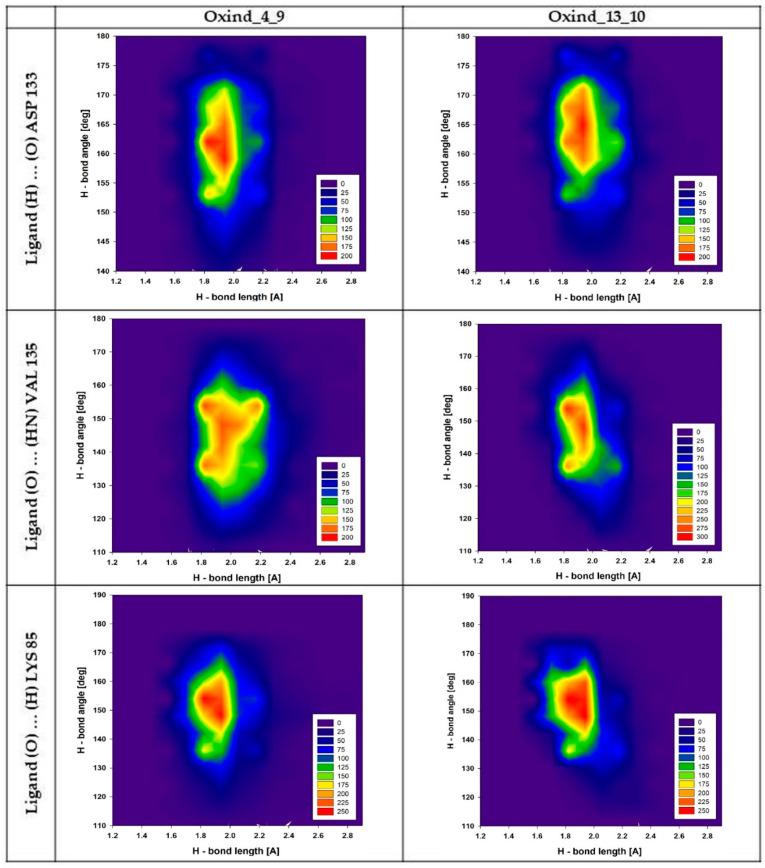
Distributions presenting mutual dependencies between values of angles and lengths characterizing hydrogen bonds created by Oxind_4_9 and Oxind_13_10 molecules with chosen amino acids from the GSK-3β active site. The headings of the individual columns define the name of the ligand, while the line descriptions define the hydrogen bond under consideration.

The best example of the first group is Oxind_13_10. The population of RMSD values describing the dynamic properties of this molecule is very uniform, which is confirmed by the plot presented in Figure 4 and by a small value of standard deviation (0.15 Å). It is also important to note that the average value of RMSD equals 0.5 Å, which indicates a very good fitting of the initial ligand structure to the protein active site obtained during the docking procedure. Very similar properties are also exhibited by Oxind_4_9 and Oxind_9_10 molecules. Oxind_4_9 is described by higher RMSD values (1.24 Å), indicating greater modification of ligand structure relative to the initial conformation; however, the distribution of data indicates significant structural stability of the obtained molecule after initial structural equilibration.

A completely different picture of dynamic structural stability can be derived from data describing Oxind_5_9 and Oxind_12_15 molecules. In the case of Oxind_5_9, despite reasonable values of average and standard deviation, analysis of the whole population distribution indicates quite significant fluctuations of RMSD values observed for ~25% of simulation time, which could indicate temporary fluctuations of the ligand structure. Oxind_12_15 exhibits the highest structural variability. This observation is confirmed not only by the value of standard deviation, which is more than twice as large as the values describing the other populations, but also by the significant fluctuations of RMSD distribution, which indicate the probable conformational instability of the ligand molecule in the GSK-3β active site. The RMSD values describing the reference molecule (3a) indicate a significant conformational stiffness of this inhibitor in the GSK-3β active site; the distribution presented in Figure S1 is uniform, and the fluctuations of presented values are slight. Among all considered oxindole derivatives, only Oxind_4_9 and Oxind_13_10 exhibit such uniform characteristics.

Another aspect that allows for determining the quality of interactions of ligands with the protein active site is the stability of interactions responsible for the stabilization of the considered complexes. The data presented in Table 4 and Figure S2 (Supplementary Materials) are the qualitative and quantitative descriptions of all hydrogen bonds identified and observed for considered complexes during the molecular dynamics stage. Not all hydrogen bonds identified during the docking procedure proved to be stable. In some cases, new interactions were observed during the molecular dynamics stage involving new amino acids. The analysis of all considered GSK-3β protein complexes confirms that in the context of interactions with oxindole derivatives, two residues, namely VAL 135 and ASP 133, play a crucial role in maintaining the stability of considered systems. In almost all analyzed systems, hydrogen bonds created by these two amino acids with atoms from ligand cores are observed for nearly all conformers collected during simulation. The majority of such interactions can be classified as medium strength hydrogen bonds (from 80 to 85% for ASP133; from 65 to 85% for VAL135). Among all complexes, there is one distinct system created by Oxind_12_15 where binding activity in this area is slightly smaller and the share of medium strength interactions significantly decreases (65% ASP133). The slight stability discrepancies of the previously presented interactions observed for compared systems manifest themselves to a much greater extent in the case of the third hydrogen bond, created by oxygen from oxindole core with hydrogen atoms from LYS85. The presence of this impact in analyzed populations of conformers collected for particular complexes ranges from ~94 to ~99%, and most of them exhibit properties indicating medium strength hydrogen bonds (from ~75 to ~80%). Significantly different is the picture of properties of this bond for complexes created by Oxind_12_15; the presence of such interaction was found only for ~44% of analyzed conformers. This observation clearly shows that the source of ligand instability during molecular dynamics may be the substituent from the side chain localized in the R1 position. The next group of factors stabilizing considered complexes, with much more differentiating binding properties of particular ligands, are interactions of functional groups localized in side chains. The Oxind_4_9 molecule creates two hydrogen bonds, both related to the activity of the substituent from the side chain localized in the R1 position. The interaction with ASN 64 identified during the docking stage disappears in the early stage of simulation and is replaced by a competitive hydrogen bond created with GLY 68 (~91%). The conformational changes of the side chain related to the appearance of interaction with GLY68 also allowed for the creation of additional hydrogen bond, which occurs in half of the population of analyzed conformers and is localized between the oxygen atom from the five-membered ring and the hydrogen atoms from LYS 85 amino group. The functional groups from side chains of the next oxindole derivative, namely Oxind_5_9, create two labile interactions with ASN 64 and PRO136, and each of them is observed in approximately 30% of considered conformers. Taking into account the frequency of appearance and measured lengths of hydrogen bonds, the impact of such interactions on the global stabilizing effect is not significant. Both side chains of Oxind_9_10 are involved in creation of hydrogen bonds. More stable interaction, observed for ~80% of analyzed conformers, is created with PRO 136. The second impact, much less stable (~45.5%), is observed between fluoride atoms and hydrogens of the amino group of LYS181. The functional group of side chains of the next ligand molecule, namely Oxind_12_15, creates only one hydrogen bond with ARG141. Such interactions are observed for 51% of considered complexes, and most of the observed impacts can be classified as weak hydrogen bonds. In the case of the last oxindole derivative, namely Oxind_13_10, two interactions are observed in which the same functional group from one side chain is involved. The hydrogen bond with TYR134 identified during the docking stage is observed for ~50% of analyzed conformers; however, conformational changes contributed to the appearance of a new interaction, namely with PRO136, which is observed for over 80% of conformers obtained during molecular dynamics simulation. The greater significance of the new impact is evidenced not only by its frequency of occurrence but also by the significant share of bonds with greater strength. The conformational changes that occurred in the active site allowed for the coexistence of both hydrogen bonds, which is the next important contribution to increasing the general stability of the considered complex. The reference molecule 3a creates only two hydrogen bonds with residues from the GSK-3β active site, namely with ASP 133 and VAL 135 (Table 4). The distributions presented in Supplementary Materials Figure S2a,b show that such interactions are stable during the whole simulation. The first hydrogen bond created with ASP 133 exhibits significant stability when compared with the rest of the considered oxindole derivatives, which is confirmed by the largest fraction of conformers characterized by the smallest hydrogen bond lengths. In the case of the second interaction, created with VAL 135, a diametrically different situation is observed; this bond is the weakest among all considered oxindole derivatives.

An important factor indicating the stability of hydrogen bonds is the fluctuations of angles and lengths noted for interacting molecules. Figure 4 presents plots describing mutual distributions of hydrogen bond lengths and angles for the most important interactions created by Oxind_4_9 and Oxind_13_10 derivatives. The first presented interaction is created by ligands with ASP 133 residue; in both cases, uniform and quite tight distributions of values are observed. The angle of hydrogen bonds of both compared interactions is placed in the range from 150 to 175°; however, in both cases, a substantial accumulation of values placed in the range not exceeding 10° can be observed, namely from 155 to 165° (Oxind_4_9) and from 160 to 170° (Oxind_13_10). The stability of this interaction is also confirmed by small fluctuations of hydrogen bond lengths. In both cases, most analyzed conformers are described by values placed in the range from 1.8 to 2.1 Å; however, in the case of bonds created by Oxind_13_10, a substantial fraction of conformers described by values exceeding 2.1 Å is observed. The next presented hydrogen bonds, created by ligands with VAL 135 residue, exhibit more diverse characteristics. For both distributions of angle values, an increase in range, which can be characterized by values from 120 to 165°, is observed. In both distributions, the highest number of conformers is characterized by values in the range from 135 to 160°. Significant differences are observed for hydrogen bond length distributions in the case of the population describing interactions created by Oxind_13_10. The majority of conformers are described by values from 1.8 to 2.1 Å, while in the case of the second considered inhibitor, such range extends from 1.8 to 2.3 Å. The last two plots presented in Figure 4 describe hydrogen bonds created by LYS 85 residue. In both cases, observed distributions are very similar. The angle values are placed in the range from 130 to 170°, and the highest concentration of values is noted between 145 and 160°. The distances characterizing presented populations of hydrogen bonds are placed in the range from 1.7 to 2.1 Å; however, the highest number of conformers adopt values from 1.8 to 2 Å.

**Figure 5:**
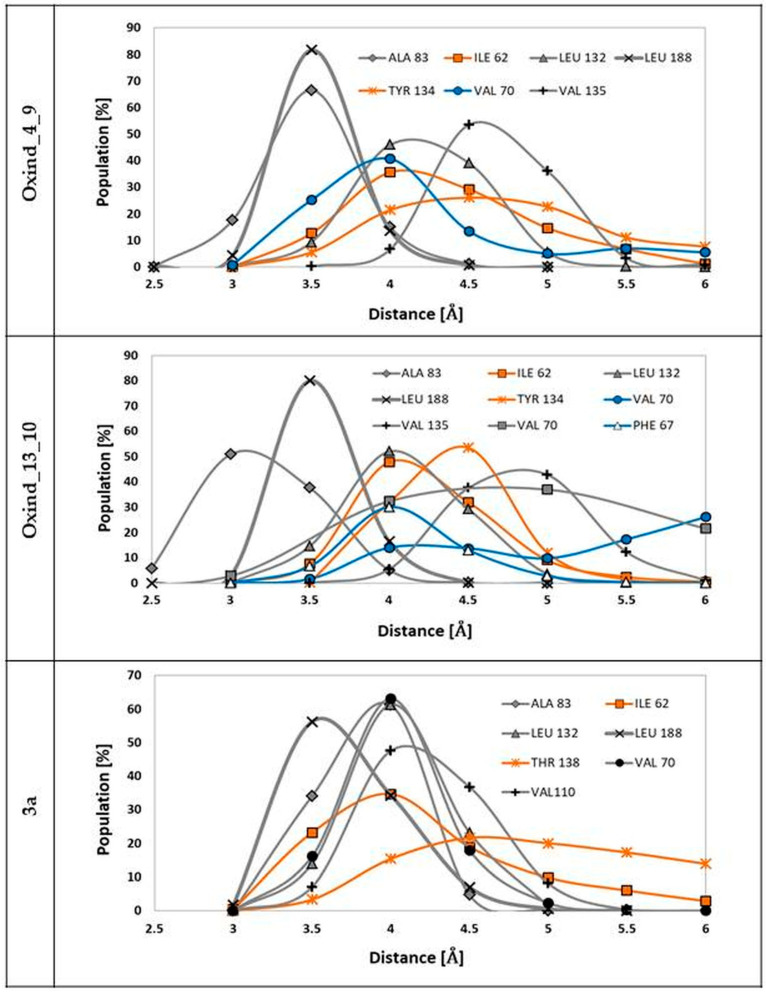
The distributions of distances measured between hydrophobic moieties of particular residues from GSK-3β active site and carbon atoms from ligand molecules. The grey distributions represent residues interacting with the molecular core, the blue distributions represent residues interacting with functional groups localized in the R1 position and the orange distributions represent residues interacting with functional groups connected to the R2 position. The markings on the left side of individual figures are the names of ligands involved in interactions presented on particular charts.

Among all forces responsible for stabilizing protein–ligand complexes, hydrophobic interactions play an important role. Numerous hydrophobic moieties are localized in the active site of the GSK-3β protein. The most important ones in the context of interactions with oxindole derivatives are LEU 132, LEU 188, VAL 135, VAL 70 and ALA 83, the localization of which provides free interactions with the oxindole core. The second group of hydrophobic moieties comprises residues interacting with functional groups substituted to positions R1 and R2, namely TYR 134, ILE 62, VAL 70 and PHE66. Figure 5 presents distributions of distances between hydrophobic moieties of particular amino acids and carbon atoms from chosen ligands. The analysis of the presented populations shows that in the case of both considered ligands, namely Oxind_4_9 and Oxind_13_10, the oxindole core is localized in the center of the hydrophobic cluster. Numerous hydrophobic interactions noted for this part of ligand molecule exhibit significant stability during the whole simulation time, as confirmed by, for example, interactions of LEU 188 and ALA 83 where nearly 100% of gathered conformations are placed in a very tight range of distances from 3 to 4 Å. In the case of the rest of the identified hydrophobic interactions involving oxindole core, the measured distances fluctuate in a larger range. The hydrophobic interactions are also involved in the stabilization of functional groups substituted to positions R1 and R2 of considered oxindole derivatives. Higher binding activity of groups localized in the R2 position, which creates quite stable interactions with TYR 134 and ILE 62, is observed in both considered cases. The conformation of the 3a molecule in the GSK-3β active site provides the occurrence of stable hydrophobic interactions of oxindole core with ALA 83, VAL 70, LEU 132, LEU 188 and VAL 110 residues; moreover, the functional groups localized in the R2 position participate in such interactions with ILE 62 and THR 134. The quite uniform distributions describing contacts between oxindole core and all hydrophobic moieties of presented residues indicate an optimal localization of the inhibitor in the space of the hydrophobic pocket.

The energetic properties of complexes considered during the molecular dynamics stage were also analyzed with the use of the MMPBSA method, which allowed for the estimation of binding enthalpies (ΔH) for populations of obtained conformers of compared complexes. The summarized data are presented in Table 5. Among all considered systems, complexes created by Oxind_4_9 and Oxind_13_10 should be distinguished. Both mentioned inhibitors exhibit the best binding capabilities towards the GSK-3β active site, which is confirmed by the values of binding enthalpy close to ~38 kcal/mol and standard deviations indicating a very similar distribution of analyzed values in the whole population of considered conformers.

The next group, showing slightly worse stability, are complexes created by Oxind_5_9 and Oxind_9_10. Among all considered inhibitors, the worst binding capabilities are exhibited by Oxind_12_15, the complexes of which were described by the lowest values of binding enthalpy and high standard deviations indicating significant energetic diversity of such systems. The CHEMBL272026 and CHEMBL410072 molecules are oxindole derivatives that were the subject of research described in previous works [35,37]. The realized calculations also included the evaluation of binding enthalpy based on the MMPBSA method for ligand complexes created with the GSK-3β active site. Obtained values of ΔH are −32.74 (±4.43) kcal/mol for CHEMBL272026 and −30.71 (±4.74) kcal/mol for CHEMBL410072. The comparison with values characterizing new oxindole derivatives clearly shows that in two cases, namely for Oxind_4_9 and Oxind_13_10, a quite significant increase in binding enthalpy is observed. Another determinant that allows for the evaluation of the obtained values characterizing individual oxindole derivatives is the comparison with the reference molecule. The obtained values show that both Oxind_4_9 and Oxind_13_10 exhibit similar binding properties to 3-(2-pyridinylmethylene)-2-oxindole.

## 4. Conclusions

The group of oxindole derivatives proposed in this paper exhibits significant affinity towards the GSK-3β active site. Presented data, obtained during molecular docking and molecular dynamics simulations, clearly show that the proposed modifications of the native molecular core significantly influenced the binding capabilities of obtained molecules. Most of the proposed active groups used in the modification of both side chains caused the assumed effects (R1–17; R2–18). The comparative analysis of both types of modifications used shows that addition in the R2 position is more favorable, which is confirmed by not only the observed increase in binding affinity values relative to the native molecule but also the number of monoderivatives that exceeded the 10% threshold. For both side chains of considered oxindole derivatives, the highest increases in binding capabilities were related to the use of active groups containing aromatic or heterocyclic rings and a set of hydrogen bond donors and recipients. The preference of such substituents indicates that the stabilization of considered complexes in this section of the active site is dependent on two types of interactions, namely hydrogen bonds and hydrophobic interactions. The chosen inhibitors, exhibiting the best binding capabilities and characterized by acceptable toxicity and good permeability through cell membranes, were also assessed with the use of molecular dynamics methods enabling the analysis of dynamic behavior of ligand–protein complexes. The data presented in this work show that not all potential inhibitors identified during preliminary analysis based on docking procedure remain stable. From five chosen complexes exhibiting primarily similar properties, only some remain stable during molecular dynamics simulation. Two complexes deserve special distinction, namely systems created by Oxind_4_9 and Oxind_13_10. In both cases, structural stability of ligand–protein complexes is confirmed by uniform distributions of RMSD values and stability of interactions observed in considered active sites during the whole simulation time. The structural stability of these complexes is also confirmed by the highest values of binding enthalpy noted for these systems. An important determinant of the potential inhibitory abilities of the tested compounds against the GSK-3β protein is their confrontation with a reference molecule (3a) with an experimentally confirmed inhibitory potential. In the case of Oxind_4_9 and Oxind_13_10, all the analyzed aspects, i.e., the structural stability of the analyzed complexes, the quantity and quality of hydrogen bonds, the hydrophobic interactions and the binding enthalpy value, clearly indicate a very similar binding potential of the compared substances.

## Figures and Tables

**Table 1:** The values of binding affinity of oxindole monoderivatives towards GSK-3β. Increase in binding affinity estimated relative to the value for Oxind_0_0, equal to −8.4 kcal/mol.

Name	Binding Affinity (kcal/mol)	Increase in Binding Affinity (%)	Name	Binding Affinity (kcal/mol)	Increase in Binding Affinity (%)
Oxind_11_0	−10.10	20.2	Oxind_0_9	−10.44	24.3
Oxind_12_0	−10.00	19.0	Oxind_0_10	−10.36	23.3
Oxind_10_0	−9.90	17.9	Oxind_0_15	−10.22	21.7
Oxind_9_0	−9.84	17.1	Oxind_0_14	−10.20	21.4
Oxind_13_0	−9.80	16.7	Oxind_0_18	−10.00	19.0
Oxind_5_0	−9.60	14.3	Oxind_0_19	−9.90	17.9
Oxind_20_0	−9.54	13.6	Oxind_0_5	−9.80	16.7
Oxind_4_0	−9.40	11.9	Oxind_0_13	−9.70	15.5
Oxind_15_0	−9.28	10.5	Oxind_0_11	−9.60	14.3
Oxind_14_0	−9.26	10.2	Oxind_0_12	−9.58	14.0
Oxind_6_0	−9.20	9.5	Oxind_0_1	−9.40	11.9
Oxind_18_0	−9.14	8.8	Oxind_0_2	−9.24	10.0
Oxind_1_0	−9.10	8.3	Oxind_0_4	−9.20	9.5
Oxind_2_0	−9.00	7.1	Oxind_0_3	−9.12	8.6
Oxind_3_0	−8.88	5.7	Oxind_0_6	−9.10	8.3
Oxind_8_0	−8.80	4.8	Oxind_0_8	−8.90	6.0
Oxind_7_0	−8.50	1.2	Oxind_0_7	−8.80	4.8
Oxind_17_0	−8.30	−1.2	Oxind_0_16	−8.60	2.4
Oxind_19_0	−8.16	−2.9	Oxind_0_17	−8.30	−1.2
Oxind_16_0	−8.00	−4.8	Oxind_0_20	−8.24	−1.9

**Table 2:** The values of binding affinity and molecular parameters for the structures exhibiting the best properties. IC/IC ref represents normalized factors describing the decrease in inhibition constant relative to the reference system (IC ref = 696.17 nM).

Name		LogP	Toxicity	Binding Affinity (kcal/mol)	Increase in Binding Affinity (%)	Inhibition Constant (nM)	IC/IC Ref
1.	Oxind_13_10	2.38	0.28	−11.60	38.10	3.14	221.6
2.	Oxind_12_15	3.07	0.38	−11.50	36.90	3.72	187.2
3.	Oxind_9_10	5.12	0.39	−11.46	36.43	3.98	175.0
4.	Oxind_4_9	3.58	0.32	−11.40	35.71	4.40	158.1
5.	Oxind_5_9	2.68	0.39	−11.22	33.57	5.97	116.7
6.	Oxind_9_15	3.64	0.19	−11.20	33.33	6.17	112.8
7.	Oxind_4_10	2.04	0.07	−11.14	32.62	6.83	102.0
8.	Oxind_15_9	2.58	0.25	−11.12	32.38	7.06	98.6
9.	Oxind_12_14	2.99	0.1	−11.10	32.14	7.30	95.3
10.	Oxind_10_14	1.82	0.04	−11.02	31.19	8.36	83.3
11.	Oxind_4_18	1.67	0.29	−10.96	30.48	9.25	75.2
12.	Oxind_20_15	2.18	0.19	−10.90	29.76	10.24	68.0
13.	Oxind_10_13	3.38	0.28	−10.90	29.76	10.24	68.0
14.	Oxind_10_18	3.43	0.03	−10.88	29.52	10.59	65.7
15.	Oxind_12_18	3.48	0.18	−10.88	29.52	10.59	65.7

**Table 3:** The average values of RMSD for ligands and enzymes for all steps used during structural analysis. The values in italics represent standard deviations (SDs). The LIG captions represent values characterizing appropriate ligand molecules in considered complexes.

	Oxind_4_9		Oxind_5_9		Oxind_9_10		Oxind_12_15		Oxind_13_10		3a	
	GSK3β	LIG	GSK3β	LIG	GSK3β	LIG	GSK3β	LIG	GSK3β	LIG	GSK3β	LIG
**RMSD**	2.47	1.24	2.42	1.62	2.28	0.77	2.61	1.97	2.25	0.50	2.37	1.14
**SD**	*0.17*	*0.14*	*0.18*	*0.25*	*0.21*	*0.21*	*0.19*	*0.54*	*0.17*	*0.15*	*0.22*	*0.13*

**Table 4:** Distribution of the most frequently created hydrogen bonds between ligand molecules and selected amino acids from the GSK-3β active site. The hydrogen bonds in the table represent middle values of intervals with a width of 0.2 Å.

Hydrogen Bond	Population%								
	∑	1.6 Å	1.8 Å	2.0 Å	2.2 Å	2.4 Å	2.6 Å	2.8 Å	3.0 Å
Oxind_4_9									
Ligand (**H3**) … (**O**) ASP 133	**100**	1.8	39.4	44.2	12.4	2.0	0.2	0.1	0,0
Ligand(**O2**) … (**HN**) VAL 135	**99.9**	0.3	19.9	48.0	22.9	6.4	1.9	0.5	0.1
Ligand (**O3**) … (**H**) LYS 85	**99.2**	2.7	43.7	37.1	10.7	3.4	1.0	0.4	0.2
Ligand (**O1**) … (**H**) LYS 85	**50.0**	0.0	0.3	1.2	3.5	6.8	10.2	14.0	14.0
Ligand (**H4**) … (**O**) ASN 64	**7.4**	0.1	1.7	2.4	1.0	0.4	0.2	0.5	1.1
Ligand (**H4**) … (**O**) GLY 68	**90.6**	0.8	26.0	36.2	16.2	6.0	2.9	1.4	0.9
Oxind_5_9									
Ligand (**H4**) … (**O**) ASP 133	**100.0**	1.6	48.1	40.2	8.6	1.1	0.3	0.1	0.0
Ligand(**O2**) … (**HN**) VAL 135	**99.8**	0.2	21.5	44.2	23.4	7.2	2.1	0.9	0.1
Ligand (**O3**) … (**H**) LYS 85	**95.1**	2.4	41.5	34.5	10.6	3.2	1.4	0.8	0.6
Ligand (**H1**) … (**O**) PRO 136	**33.8**	0.2	6.2	12.2	6.4	4.1	1.7	1.5	1.5
Ligand (**H2**) … (**O**) ASN 64	**25.0**	0.1	2.4	5.6	4.9	3.3	2.9	2.4	3.4
Oxind_9_10									
Ligand (**H3**) … (**O**) ASP 133	**100.0**	1.4	34.1	46.0	14.4	3.4	0.6	0.0	0.1
Ligand(**O2**) … (**HN**) VAL 135	**100.0**	0.3	26.6	48.7	19.3	4.3	0.7	0.0	0.1
Ligand (**O2**) … (**H**) LYS 85	**99.3**	4.2	49.7	34.7	7.3	2.4	0.6	0.2	0.2
Ligand (**Fx**) … (**H**) LYS 181	**45.4**	0.0	0.0	0.9	3.3	6.4	11.4	11.4	12.1
Ligand (**H3**) … (**O**) PRO 136	**79.9**	1.6	10.6	18.6	16.2	13.5	8.5	6.3	4.6
Oxind_12_15									
Ligand (**H3**) … (**O**) ASP 133	**95.6**	0.1	21.7	41.9	20.4	7.4	2.7	0.8	0.7
Ligand(**O3**) … (**HN**) VAL 135	**98.3**	0.8	25.3	42.9	21.4	5.1	1.7	0.5	0.8
Ligand (**O4**) … (**H**) LYS 85	**43.9**	0.3	13.0	15.9	7.8	3.1	1.9	1.3	0.7
Ligand (**N1**) … (**H**) ARG 141	**51.0**	0.0	2.9	13.0	11.8	7.8	6.4	4.1	5.1
Oxind_13_10									
Ligand (**H3**) … (**O**) ASP 133	**100.0**	0.6	29.2	48.6	18.1	2.6	0.8	0.2	0.0
Ligand(**O1**) … (**HN**) VAL 135	**100.0**	0.6	34.2	49.5	13.3	2.2	0.2	0.0	0.0
Ligand (**O2**) … (**H**) LYS 85	**93.9**	3.8	46.5	31.6	8.1	2.9	0.5	0.2	0.3
Ligand (**H2**) … (**O**) PRO 136	**80.3**	6.3	24.4	21.9	11.5	6.4	4.0	2.9	2.9
Ligand (**H2**) … (**O**) TYR 134	**50.1**	0.0	0.7	1.7	3.5	5.6	7.6	13.7	17.4
3a									
Ligand (**H3**) … (**O**) ASP 133	**100**	2.5	55.6	36.0	5.1	0.9	0.0	0.0	0.0
Ligand(**O1**) … (**HN**) VAL 135	**96.5**	0.5	18.2	35.9	22.1	10.3	4.1	2.7	2.7

**Table 5:** The values of binding enthalpy ΔH (kcal/mol) estimated for GSK-3β complexes considered during the molecular dynamics stage (E_VDWAALS_ = van der Waals contribution from MM; E_EL_ = electrostatic energy; E_PB_ = the electrostatic contribution to the solvation free energy calculated by PB; E_CAVITY_ = nonpolar contribution to the solvation free energy; ΔH = final estimated binding enthalpy).

	Oxind_4_9		Oxind_5_9		Oxind_9_10		Oxind_12_15		Oxind_13_10		3a	
	ΔE	SD	ΔE	SD	ΔE	SD	ΔE	SD	ΔE	SD	ΔE	SD
**E_VDWAALS_**	−48.31	2.63	−45.52	3.21	−50.63	3.79	−46.56	6.62	−49.80	2.98	−47.91	2.76
**E_EL_**	−51.29	3.88	−49.62	4.95	−49.80	5.20	−47.52	7.38	−50.85	3.66	−49.84	3.67
**E_PB_**	65.76	4.94	67.84	6.89	70.68	5.97	68.54	9.50	66.19	4.38	65.21	4.15
**E_CAVITY_**	−3.97	1.15	−4.29	1.66	−3.95	1.96	−3.58	1.99	−4.17	1.59	−3.85	1.35
**ΔH**	**−37.80**	**6.91**	**−31.59**	**9.22**	**−33.71**	**8.99**	**−29.13**	**13.87**	**−38.63**	**6.63**	**−36.39**	**6.33**

## Data Availability

Data are contained within the article or Supplementary Materials.

## Supplementary Materials

The following are available online at https://www.mdpi.com/article/10.3390/biology10040332/s1. Figure S1: Distributions of RMSD values. Black colour refers to GSK-3β protein while gray refers to ligand molecules. Figure S2: Distributions of the most frequently created hydrogen bonds between ligand molecules and selected amino acids from the GSK-3β active site (a) ASP133, (b) VAL135, (c) LYS85. Table S1: The characteristics of affinities and molecular descriptors of analysed oxindole derivatives. (ΔG—ligand binding affinity; IC—Inhibition constant; Tox—index of toxicity; logP –octanol/water coefficient; MW—molecular weight; n HbA- number of hydrogen bond acceptors; n HbD—number of hydrogen bond donors.) Part of the data covering the molecular properties of chosen derivatives was previously published in the work dedicated to inhibition of the CDK2 enzyme. Table S2: Interactions of oxindole derivatives with amino acids from GSK-3β active site, estimated during docking stage.
