# Supplementary material for: The Oxindole Derivatives, New Promising GSK-3β Inhibitors as One of the Potential Treatments for Alzheimer’s Disease—A Molecular Dynamics Approach
Source: Biology (Basel). 2021 Apr 15;10(4):332. doi: 10.3390/biology10040332 (PMC8071161; doi:10.3390/biology10040332)
Supplement: Supplementary file 1 [file biology-10-00332-s001.zip › biology-1176197-supplementary.pdf]

The Oxindole Derivatives, new Promising GSK-3β Inhibitors as One of the Potential Treatments for Alzheimer's Disease – a Molecular Dynamics Approach

Przemysław Czeleń[a*], and Beata Szefler[a]

[a]Department of Physical Chemistry, Faculty of Pharmacy, Collegium Medicum, Nicolaus Copernicus University, Kurpinskiego 5, 85-096 Bydgoszcz, Poland

* Correspondence: Przemysław Czeleń przemekcz@cm.umk.pl

Supplementary Materials

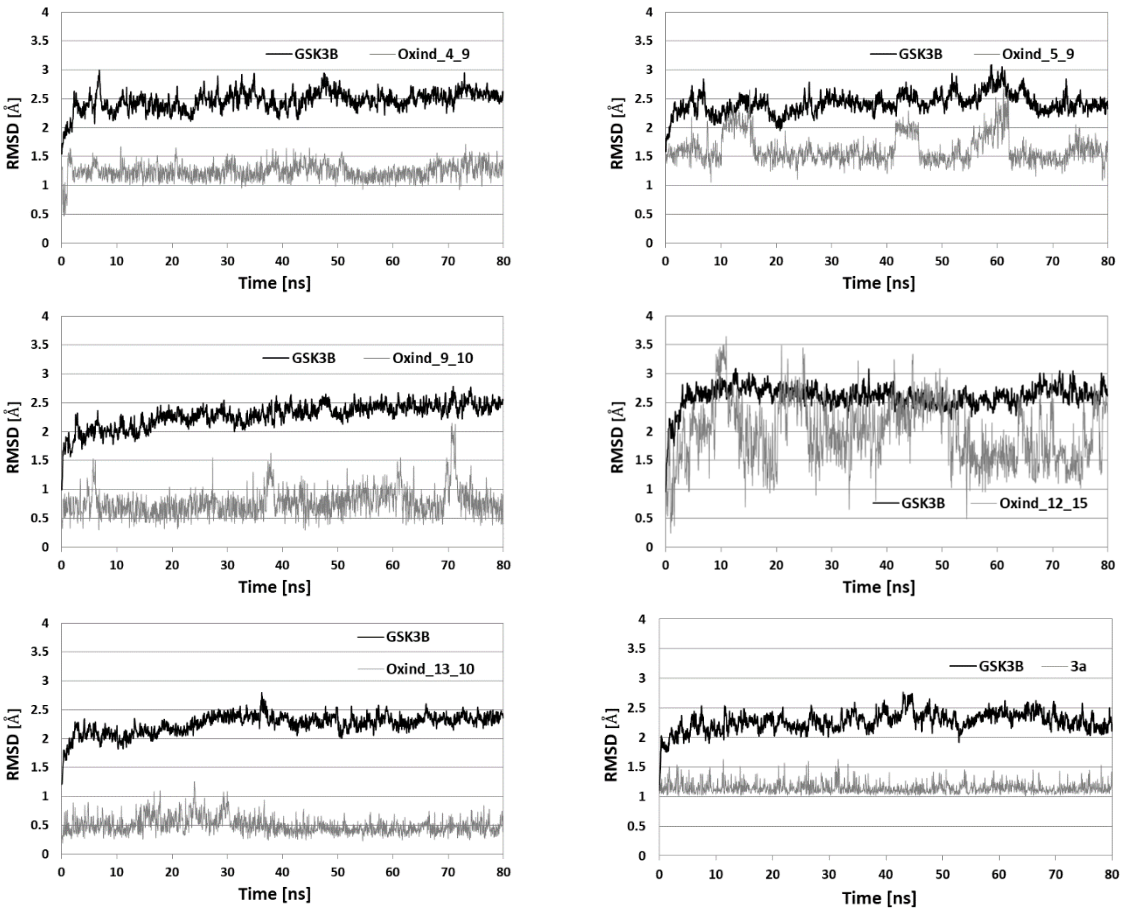

Figure S1. Distributions of RMSD values. Black colour refers to GSK-3β protein while gray refers to ligand molecules. .

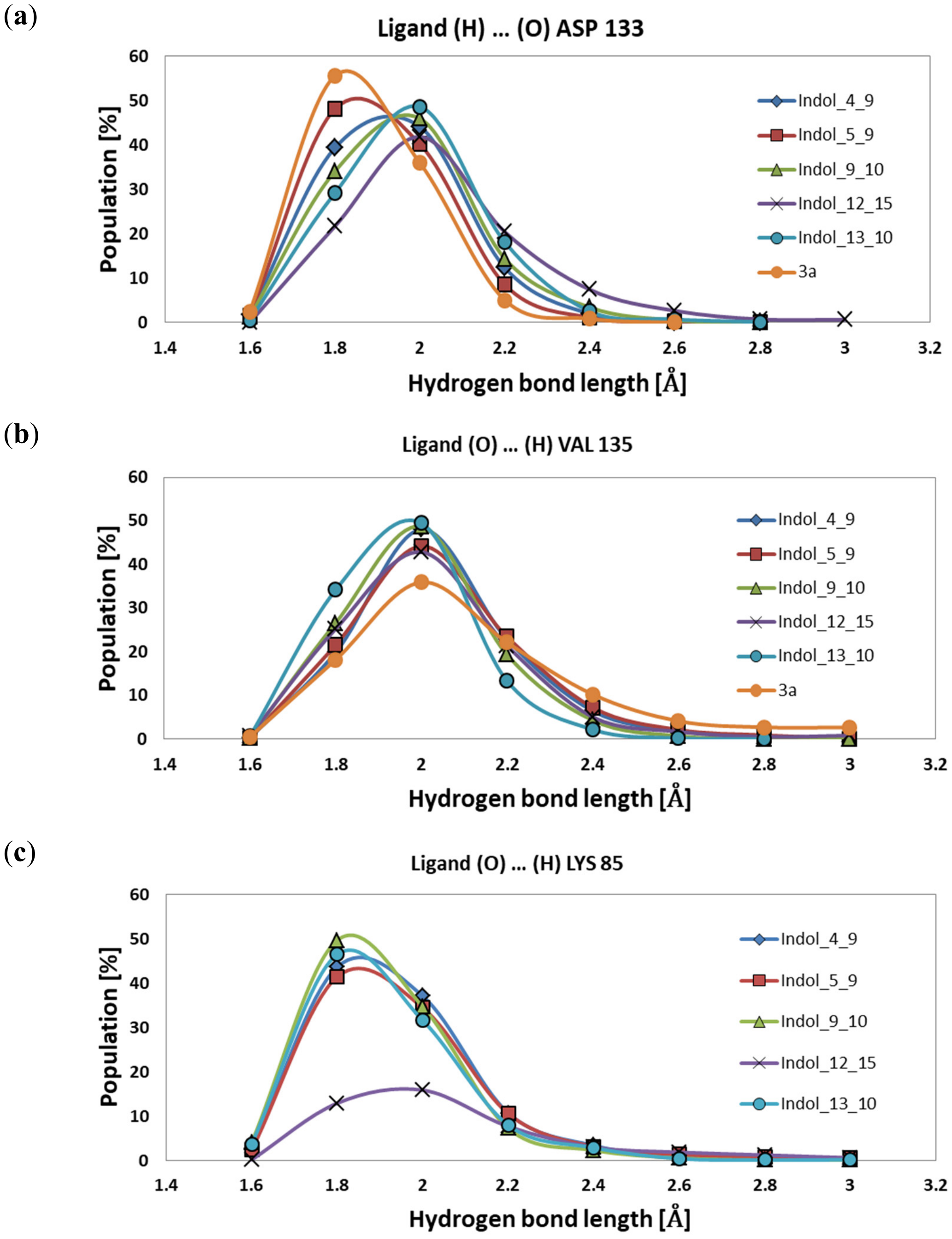

Figure S2. Distributions of the most frequently created hydrogen bonds between ligand molecules and selected amino acids from the GSK-3β active site (**a**)ASP133, (**b**) VAL135, (**c**) LYS85.

Table S2. Interactions of oxindole derivatives with amino acids from GSK-3β active site, estimated during docking stage.

Amino Acid	Hydrogen-Bond Length				
	**Oxind_4_9**	**Oxind_5_9**	**Oxind_9_10**	**Oxind_12_15**	**Oxind_13_10**
ASP 133	2.4	1.97	2.19	2.57	2.41
VAL 135	2.02	1.82	1.94	2.27	2.07
LYS 85	2.42	2.46	2.24	2.18	2.21
ASN 64	2.73	1.93	---	---	2.37
THR 138	2.85	2.96	---	---	---
ARG 141	3.07	2.86	3.02	2.65 ; 2.76	2.88
PRO 136	---	---	2.4	---	---
LYS 183	---	---	2.29 ; 2.66	---	---
THR 134	---	---	---	---	2.23

Table S1. The characteristics of affinities and molecular descriptors of analysed oxindole derivatives. (ΔG –ligand binding affinity; IC –Inhibition constant ; Tox – index of toxicity; logP –octanol/water coefficient; MW – molecular weight; n HbA- number of hydrogen bond acceptors; n HbD – number of hydrogen bond donors.) Part of the data covering the molecular properties of chosen derivatives was previously published in the work dedicated to inhibition of the CDK2 enzyme [21]

Name	SMILES	ΔG [kcal/mol]	IC [nM]	logP	Tox	MW [g/mol]	n HbA	n HbD
Oxind_4_1	c12c(cc(cc1)C(=O)NCn1c(=O)[nH]cc1)/C(=C/CCc1nonc1N)/C(=O)N2	-10.36	25.47	0.31	0.97	395.38	11	5
Oxind_4_2	c12c(cc(cc1)C(=O)NCn1c(=O)[nH]cc1)/C(=C/CCCc1nc[nH]c1)/C(=O)N2	-10	46.76	1.07	0.79	392.42	9	4
Oxind_4_5	c12c(cc(cc1)C(=O)NCn1c(=O)[nH]cc1)/C(=C/CC1=CC(=NC1=O)NC)/C(=O)N2	-10.6	16.99	-1.32	0.43	406.4	10	4
Oxind_4_9	c12c(cc(cc1)C(=O)NCn1c(=O)[nH]cc1)/C(=C/Cc1cc(cc(c1)NC)C(F)(F)F)/C(=O)N2	-11.4	4.40	3.58	0.32	471.44	8	4
Oxind_4_10	c12c(cc(cc1)C(=O)NCn1c(=O)[nH]cc1)/C(=C/Cc1cc(ccc1)C(=O)O)/C(=O)N2	-11.14	6.83	2.04	0.07	418.41	9	4
Oxind_4_11	c12c(cc(cc1)C(=O)NCn1c(=O)[nH]cc1)/C(=C/Cc1ccnc(n1)N)/C(=O)N2	-10.58	17.57	-0.01	0.88	391.39	10	5
Oxind_4_12	c12c(cc(cc1)C(=O)NCn1c(=O)[nH]cc1)/C(=C/CNc1ccc(cc1)Br)/C(=O)N2	-10.52	19.44	2.80	0.35	468.31	8	4
Oxind_4_13	c12c(cc(cc1)C(=O)NCn1c(=O)[nH]cc1)/C(=C/CC1CCNCC1)/C(=O)N2	-10.46	21.51	1.41	0.17	381.44	8	4
Oxind_4_14	c12c(cc(cc1)C(=O)NCn1c(=O)[nH]cc1)/C(=C/CC1CCN(CC1)S(=O)(=O)C)/C(=O)N2	-10.78	12.54	0.12	0.01	459.53	10	3
Oxind_4_15	c12c(cc(cc1)C(=O)NCn1c(=O)[nH]cc1)/C(=C/CCc1ccc(cc1)S(=O)(=O)N)/C(=O)N2	-10.8	12.12	0.45	0.01	467.51	10	5
Oxind_4_18	c12c(cc(cc1)C(=O)NCn1c(=O)[nH]cc1)/C(=C/CN(C(=O)Nc1ccccc1)C)/C(=O)N2	-10.96	9.25	1.67	0.29	446.47	10	4
Oxind_4_19	c12c(cc(cc1)C(=O)NCn1c(=O)[nH]cc1)/C(=C/CNC(=O)c1ccccc1)/C(=O)N2	-10.34	26.34	0.85	0.00	417.43	9	4
Oxind_5_1	c12c(cc(cc1)C(=O)NCC1=CC(=NC1=O)NC)/C(=C/CCc1nonc1N)/C(=O)N2	-10.5	20.11	-1.24	0.93	421.42	11	5
Oxind_5_2	c12c(cc(cc1)C(=O)NCC1=CC(=NC1=O)NC)/C(=C/CCCc1nc[nH]c1)/C(=O)N2	-10.14	36.92	-0.34	0.69	418.46	9	4
Oxind_5_9	c12c(cc(cc1)C(=O)NCC1=CC(=NC1=O)NC)/C(=C/Cc1cc(cc(c1)NC)C(F)(F)F)/C(=O)N2	-11.22	5.97	2.68	0.39	497.48	8	4
Oxind_5_10	c12c(cc(cc1)C(=O)NCC1=CC(=NC1=O)NC)/C(=C/Cc1cc(ccc1)C(=O)O)/C(=O)N2	-11.1	7.30	0.95	0.09	444.45	9	4
Oxind_5_11	c12c(cc(cc1)C(=O)NCC1=CC(=NC1=O)NC)/C(=C/Cc1ccnc(n1)N)/C(=O)N2	-10.46	21.51	-0.67	0.68	417.43	10	5
Oxind_5_12	c12c(cc(cc1)C(=O)NCC1=CC(=NC1=O)NC)/C(=C/CNc1ccc(cc1)Br)/C(=O)N2	-10.6	16.99	1.89	0.43	494.35	8	4
Oxind_5_13	c12c(cc(cc1)C(=O)NCC1=CC(=NC1=O)NC)/C(=C/CC1CCNCC1)/C(=O)N2	-10.7	14.35	0.87	0.23	407.47	8	4
Oxind_5_14	c12c(cc(cc1)C(=O)NCC1=CC(=NC1=O)NC)/C(=C/CC1CCN(CC1)S(=O)(=O)C)/C(=O)N2	-10.4	23.81	-0.77	0.01	485.57	10	3
Oxind_5_15	c12c(cc(cc1)C(=O)NCC1=CC(=NC1=O)NC)/C(=C/CCc1ccc(cc1)S(=O)(=O)N)/C(=O)N2	-11.1	7.30	-0.22	0.01	493.55	10	5
Oxind_5_18	c12c(cc(cc1)C(=O)NCC1=CC(=NC1=O)NC)/C(=C/CN(C(=O)Nc1ccccc1)C)/C(=O)N2	-11.3	5.21	0.84	0.31	472.5	10	4
Oxind_5_19	c12c(cc(cc1)C(=O)NCC1=CC(=NC1=O)NC)/C(=C/CNC(=O)c1ccccc1)/C(=O)N2	-10.1	39.50	0.45	0.00	443.46	9	4
Oxind_9_1	c12c(cc(cc1)C(=O)NCc1cc(cc(c1)NC)C(F)(F)F)/C(=C/CCc1nonc1N)/C(=O)N2	-10.3	28.18	3.56	0.88	486.45	9	5

Oxind_9_2	c12c(cc(cc1)C(=O)NCc1cc(cc(c1)NC)C(F)(F)F)/C(=C/CCCc1nc[nH]c1)/C(=O)N2	-10.24	31.19	4.21	0.70	483.49	7	4
Oxind_9_5	c12c(cc(cc1)C(=O)NCc1cc(cc(c1)NC)C(F)(F)F)/C(=C/CC1=CC(=NC1=O)NC)/C(=O)N2	-10.8	12.12	2.07	0.36	497.48	8	4
Oxind_9_10	c12c(cc(cc1)C(=O)NCc1cc(cc(c1)NC)C(F)(F)F)/C(=C/Cc1cc(ccc1)C(=O)O)/C(=O)N2	-11.46	3.98	5.12	0.39	509.48	7	4
Oxind_9_11	c12c(cc(cc1)C(=O)NCc1cc(cc(c1)NC)C(F)(F)F)/C(=C/Cc1ccnc(n1)N)/C(=O)N2	-10.8	12.12	3.28	0.69	482.47	8	5
Oxind_9_12	c12c(cc(cc1)C(=O)NCc1cc(cc(c1)NC)C(F)(F)F)/C(=C/CNc1ccc(cc1)Br)/C(=O)N2	-10.38	24.62	5.90	0.59	559.39	6	4
Oxind_9_13	c12c(cc(cc1)C(=O)NCc1cc(cc(c1)NC)C(F)(F)F)/C(=C/CC1CCNCC1)/C(=O)N2	-10.9	10.24	4.81	0.45	472.51	6	4
Oxind_9_14	c12c(cc(cc1)C(=O)NCc1cc(cc(c1)NC)C(F)(F)F)/C(=C/CC1CCN(CC1)S(=O)(=O)C)/C(=O)N2	-10.58	17.57	3.68	0.05	550.6	8	3
Oxind_9_15	c12c(cc(cc1)C(=O)NCc1cc(cc(c1)NC)C(F)(F)F)/C(=C/CCc1ccc(cc1)S(=O)(=O)N)/C(=O)N2	-11.2	6.17	3.64	0.19	558.58	8	5
Oxind_9_18	c12c(cc(cc1)C(=O)NCc1cc(cc(c1)NC)C(F)(F)F)/C(=C/CN(C(=O)Nc1ccccc1)C)/C(=O)N2	-10.78	12.54	4.28	0.21	537.54	8	4
Oxind_9_19	c12c(cc(cc1)C(=O)NCc1cc(cc(c1)NC)C(F)(F)F)/C(=C/CNC(=O)c1ccccc1)/C(=O)N2	-9.9	55.36	4.08	0.11	508.5	7	4
Oxind_10_1	c12c(cc(cc1)C(=O)NCc1cc(ccc1)C(=O)O)/C(=C/CCc1nonc1N)/C(=O)N2	-9.92	53.52	2.24	0.79	433.42	10	5
Oxind_10_2	c12c(cc(cc1)C(=O)NC[C@@H]1C[C@H](CCC1)C(=O)O)/C(=C/CCCc1nc[nH]c1)/C(=O)N2	-10.3	28.18	2.75	0.49	436.51	8	4
Oxind_10_5	c12c(cc(cc1)C(=O)NCc1cc(ccc1)C(=O)O)/C(=C/CC1=CC(=NC1=O)NC)/C(=O)N2	-10.88	10.59	0.75	0.15	444.45	9	4
Oxind_10_9	c12c(cc(cc1)C(=O)NCc1cc(ccc1)C(=O)O)/C(=C/Cc1cc(cc(c1)NC)C(F)(F)F)/C(=O)N2	-11.34	4.87	5.27	0.46	509.48	7	4
Oxind_10_11	c12c(cc(cc1)C(=O)NCc1cc(ccc1)C(=O)O)/C(=C/Cc1ccnc(n1)N)/C(=O)N2	-10.7	14.35	1.66	0.40	429.44	9	5
Oxind_10_12	c12c(cc(cc1)C(=O)NCc1cc(ccc1)C(=O)O)/C(=C/CNc1ccc(cc1)Br)/C(=O)N2	-10.6	16.99	4.50	0.51	506.36	7	4
Oxind_10_13	c12c(cc(cc1)C(=O)NCc1cc(ccc1)C(=O)O)/C(=C/CC1CCNCC1)/C(=O)N2	-10.9	10.24	3.38	0.28	419.48	7	4
Oxind_10_14	c12c(cc(cc1)C(=O)NC[C@@H]1C[C@H](CCC1)C(=O)O)/C(=C/CC1CCN(CC1)S(=O)(=O)C)/C(=O)N2	-11.02	8.36	1.82	0.04	503.62	9	3
Oxind_10_15	c12c(cc(cc1)C(=O)NCc1cc(ccc1)C(=O)O)/C(=C/CCc1ccc(cc1)S(=O)(=O)N)/C(=O)N2	-11.22	5.97	2.32	0.45	505.55	9	5
Oxind_10_18	c12c(cc(cc1)C(=O)NC[C@@H]1C[C@H](CCC1)C(=O)O)/C(=C/CN(C(=O)Nc1ccccc1)C)/C(=O)N2	-10.88	10.59	3.43	0.03	490.56	9	4
Oxind_10_19	c12c(cc(cc1)C(=O)NCc1cc(ccc1)C(=O)O)/C(=C/CNC(=O)c1ccccc1)/C(=O)N2	-10.4	23.81	2.94	0.03	455.47	8	4
Oxind_11_1	c12c(cc(cc1)C(=O)NCc1ccnc(n1)N)/C(=C/CCc1nonc1N)/C(=O)N2	-10.46	21.51	-0.42	0.94	406.41	11	6
Oxind_11_2	c12c(cc(cc1)C(=O)NCc1ccnc(n1)N)/C(=C/CCCc1nc[nH]c1)/C(=O)N2	-10.5	20.11	1.11	0.88	403.45	9	5
Oxind_11_5	c12c(cc(cc1)C(=O)NCc1ccnc(n1)N)/C(=C/CC1=CC(=NC1=O)NC)/C(=O)N2	-11	8.65	-1.61	0.72	417.43	10	5
Oxind_11_9	c12c(cc(cc1)C(=O)NCc1ccnc(n1)N)/C(=C/Cc1cc(cc(c1)NC)C(F)(F)F)/C(=O)N2	-11.4	4.40	3.53	0.72	482.47	8	5
Oxind_11_10	c12c(cc(cc1)C(=O)NCc1ccnc(n1)N)/C(=C/Cc1cc(ccc1)C(=O)O)/C(=O)N2	-11.04	8.08	1.94	0.45	429.44	9	5
Oxind_11_12	c12c(cc(cc1)C(=O)NCc1ccnc(n1)N)/C(=C/CNc1ccc(cc1)Br)/C(=O)N2	-10.94	9.57	2.76	0.76	479.34	8	5
Oxind_11_13	c12c(cc(cc1)C(=O)NCc1ccnc(n1)N)/C(=C/CC1CCNCC1)/C(=O)N2	-11.12	7.06	1.56	0.68	392.46	8	5
Oxind_11_14	c12c(cc(cc1)C(=O)NCc1ccnc(n1)N)/C(=C/CC1CCN(CC1)S(=O)(=O)C)/C(=O)N2	-11.5	3.72	0.26	0.66	470.56	10	4

Oxind_11_15	c12c(cc(cc1)C(=O)NCc1ccnc(n1)N)/C(=C/CCc1ccc(cc1)S(=O)(=O)N)/C(=O)N2	-11.4	4.40	0.43	0.26	478.53	10	6
Oxind_11_18	c12c(cc(cc1)C(=O)NCc1ccnc(n1)N)/C(=C/CN(C(=O)Nc1ccccc1)C)/C(=O)N2	-11.2	6.17	1.79	0.89	457.49	10	5
Oxind_11_19	c12c(cc(cc1)C(=O)NCc1ccnc(n1)N)/C(=C/CNC(=O)c1ccccc1)/C(=O)N2	-11.08	7.56	0.77	0.37	428.45	9	5
Oxind_12_1	c12c(cc(cc1)C(=O)Nc1ccc(cc1)Br)/C(=C/CCc1nonc1N)/C(=O)N2	-10.8	12.12	2.19	0.85	454.28	8	4
Oxind_12_2	c12c(cc(cc1)C(=O)Nc1ccc(cc1)Br)/C(=C/CCCc1nc[nH]c1)/C(=O)N2	-10.4	23.81	3.54	0.66	451.32	6	3
Oxind_12_5	c12c(cc(cc1)C(=O)Nc1ccc(cc1)Br)/C(=C/CC1=CC(=NC1=O)NC)/C(=O)N2	-11.1	7.30	1.37	0.28	465.31	7	3
Oxind_12_9	c12c(cc(cc1)C(=O)Nc1ccc(cc1)Br)/C(=C/Cc1cc(cc(c1)NC)C(F)(F)F)/C(=O)N2	-11.98	1.65	6.03	0.66	530.34	5	3
Oxind_12_10	c12c(cc(cc1)C(=O)Nc1ccc(cc1)Br)/C(=C/Cc1cc(ccc1)C(=O)O)/C(=O)N2	-11.9	1.89	4.37	0.59	477.31	6	3
Oxind_12_11	c12c(cc(cc1)C(=O)Nc1ccc(cc1)Br)/C(=C/Cc1ccnc(n1)N)/C(=O)N2	-11.14	6.83	2.39	0.57	450.3	7	4
Oxind_12_13	c12c(cc(cc1)C(=O)Nc1ccc(cc1)Br)/C(=C/CC1CCNCC1)/C(=O)N2	-11.2	6.17	4.29	0.55	440.34	5	3
Oxind_12_14	c12c(cc(cc1)C(=O)Nc1ccc(cc1)Br)/C(=C/CC1CCN(CC1)S(=O)(=O)C)/C(=O)N2	-11.1	7.30	2.99	0.10	518.43	7	2
Oxind_12_15	c12c(cc(cc1)C(=O)Nc1ccc(cc1)Br)/C(=C/CCc1ccc(cc1)S(=O)(=O)N)/C(=O)N2	-11.5	3.72	3.07	0.38	526.41	7	4
Oxind_12_18	c12c(cc(cc1)C(=O)Nc1ccc(cc1)Br)/C(=C/CN(C(=O)Nc1ccccc1)C)/C(=O)N2	-10.88	10.59	3.48	0.18	505.37	7	3
Oxind_12_19	c12c(cc(cc1)C(=O)Nc1ccc(cc1)Br)/C(=C/CNC(=O)c1ccccc1)/C(=O)N2	-10.66	15.35	3.47	0.16	476.33	6	3
Oxind_13_1	c12c(cc(cc1)C(=O)NC1CCNCC1)/C(=C/CCc1nonc1N)/C(=O)N2	-10.6	16.99	0.28	0.88	382.42	9	5
Oxind_13_2	c12c(cc(cc1)C(=O)NC1CCNCC1)/C(=C/CCCc1nc[nH]c1)/C(=O)N2	-10.16	35.70	1.49	0.65	379.46	7	4
Oxind_13_5	c12c(cc(cc1)C(=O)NC1CCNCC1)[C@H](C(=O)N2)CCC1=CC(=NC1=O)NC	-11.1	7.30	-0.78	0.24	395.46	8	4
Oxind_13_9	c12c(cc(cc1)C(=O)NC1CCNCC1)/C(=C/Cc1cc(cc(c1)NC)C(F)(F)F)/C(=O)N2	-11.7	2.65	4.26	0.53	458.48	6	4
Oxind_13_10	c12c(cc(cc1)C(=O)NC1CCNCC1)/C(=C/Cc1cc(ccc1)C(=O)O)/C(=O)N2	-11.6	3.14	2.38	0.28	405.45	7	4
Oxind_13_11	c12c(cc(cc1)C(=O)NC1CCNCC1)/C(=C/Cc1ccnc(n1)N)/C(=O)N2	-10.9	10.24	0.69	0.64	378.44	8	5
Oxind_13_12	c12c(cc(cc1)C(=O)NC1CCNCC1)/C(=C/CNc1ccc(cc1)Br)/C(=O)N2	-10.8	12.12	3.37	0.51	455.36	6	4
Oxind_13_14	c12c(cc(cc1)C(=O)NC1CCNCC1)/C(=C/CC1CCN(CC1)S(=O)(=O)C)/C(=O)N2	-10.9	10.24	1.33	0.02	446.57	8	3
Oxind_13_15	c12c(cc(cc1)C(=O)NC1CCNCC1)/C(=C/CCc1ccc(cc1)S(=O)(=O)N)/C(=O)N2	-11.3	5.21	1.28	0.11	454.55	8	5
Oxind_13_18	c12c(cc(cc1)C(=O)NC1CCNCC1)/C(=C/CN(C(=O)Nc1ccccc1)C)/C(=O)N2	-10.8	12.12	2.37	0.16	433.51	8	4
Oxind_13_19	c12c(cc(cc1)C(=O)NC1CCNCC1)/C(=C/CNC(=O)c1ccccc1)/C(=O)N2	-10.38	24.62	1.36	0.05	404.47	7	4
Oxind_14_1	S(=O)(=O)(C)N1CCC(NC(=O)c2cc3c(cc2)NC(=O)/C/3=C\CCc2nonc2N)CC1	-9.66	83.01	-0.39	0.96	460.52	11	4
Oxind_14_2	S(=O)(=O)(C)N1CCC(NC(=O)c2cc3c(cc2)NC(=O)/C/3=C\CCCc2nc[nH]c2)CC1	-9.66	83.01	1.45	0.49	457.56	9	3
Oxind_14_5	S(=O)(=O)(C)N1CCC(NC(=O)c2cc3c(cc2)NC(=O)/C/3=C\CC2=CC(=NC2=O)NC)CC1	-9.8	65.54	-1.32	0.02	471.54	10	3
Oxind_14_9	S(=O)(=O)(C)N1CCC(NC(=O)c2cc3c(cc2)NC(=O)/C/3=C\Cc2cc(cc(c2)NC)C(F)(F)F)CC1	-10.84	11.33	3.48	0.13	536.58	8	3
Oxind_14_10	S(=O)(=O)(C)N1CCC(NC(=O)c2cc3c(cc2)NC(=O)/C/3=C\Cc2cc(ccc2)C(=O)O)CC1	-10.72	13.87	1.79	0.05	483.55	9	3

Oxind_14_11	S(=O)(=O)(C)N1CCC(NC(=O)c2cc3c(cc2)NC(=O)/C/3=C\Cc2ccnc(n2)N)CC1	-10.34	26.34	-0.04	0.63	456.53	10	4
Oxind_14_12	S(=O)(=O)(C)N1CCC(NC(=O)c2cc3c(cc2)NC(=O)/C/3=C\CNc2ccc(cc2)Br)CC1	-10.12	38.19	2.64	0.15	533.45	8	3
Oxind_14_13	S(=O)(=O)(C)N1CCC(NC(=O)c2cc3c(cc2)NC(=O)/C/3=C\CC2CCNCC2)CC1	-10.34	26.34	1.45	0.02	446.57	8	3
Oxind_14_15	S(=O)(=O)(C)N1CCC(NC(=O)c2cc3c(cc2)NC(=O)/C/3=C\CCc2ccc(cc2)S(=O)(=O)N)CC1	-10.24	31.19	0.65	0.00	532.64	10	4
Oxind_14_18	S(=O)(=O)(C)N1CCC(NC(=O)c2cc3c(cc2)NC(=O)/C/3=C\CN(C(=O)Nc2ccccc2)C)CC1	-9.2	180.43	1.73	0.00	511.6	10	3
Oxind_14_19	S(=O)(=O)(C)N1CCC(NC(=O)c2cc3c(cc2)NC(=O)/C/3=C\CNC(=O)c2ccccc2)CC1	-9.66	83.01	0.70	0.00	482.56	9	3
Oxind_15_1	c12c(cc(cc1)C(=O)NCc1ccc(cc1)S(=O)(=O)N)/C(=C/CCc1nonc1N)/C(=O)N2	-10.1	39.50	-1.34	0.87	468.5	11	6
Oxind_15_2	c12c(cc(cc1)C(=O)NCc1ccc(cc1)S(=O)(=O)N)/C(=C/CCCc1nc[nH]c1)/C(=O)N2	-10.5	20.11	0.28	0.29	465.54	9	5
Oxind_15_5	c12c(cc(cc1)C(=O)NCc1ccc(cc1)S(=O)(=O)N)/C(=C/CC1=CC(=NC1=O)NC)/C(=O)N2	-10.76	12.97	-2.23	0.02	479.52	10	5
Oxind_15_9	c12c(cc(cc1)C(=O)NCc1ccc(cc1)S(=O)(=O)N)/C(=C/Cc1cc(cc(c1)NC)C(F)(F)F)/C(=O)N2	-11.12	7.06	2.58	0.25	544.55	8	5
Oxind_15_10	c12c(cc(cc1)C(=O)NCc1ccc(cc1)S(=O)(=O)N)/C(=C/Cc1cc(ccc1)C(=O)O)/C(=O)N2	-11.12	7.06	1.00	0.49	491.52	9	5
Oxind_15_11	c12c(cc(cc1)C(=O)NCc1ccc(cc1)S(=O)(=O)N)/C(=C/Cc1ccnc(n1)N)/C(=O)N2	-10.48	20.80	-0.85	0.24	464.51	10	6
Oxind_15_12	c12c(cc(cc1)C(=O)NCc1ccc(cc1)S(=O)(=O)N)/C(=C/CNc1ccc(cc1)Br)/C(=O)N2	-10.3	28.18	1.95	0.30	541.43	8	5
Oxind_15_13	c12c(cc(cc1)C(=O)NCc1ccc(cc1)S(=O)(=O)N)/C(=C/CC1CCNCC1)/C(=O)N2	-10.64	15.88	0.66	0.14	454.55	8	5
Oxind_15_14	c12c(cc(cc1)C(=O)NCc1ccc(cc1)S(=O)(=O)N)/C(=C/CC1CCN(CC1)S(=O)(=O)C)/C(=O)N2	-11.2	6.17	-0.79	0.00	532.64	10	4
Oxind_15_18	C12C(CC(CC1)C(=O)NCc1ccc(cc1)S(=O)(=O)N)/C(=C/CN(C(=O)NC1CCCCC1)C)/C(=O)N2	-11.54	3.48	0.77	0.00	531.68	10	5
Oxind_15_19	c12c(cc(cc1)C(=O)NCc1ccc(cc1)S(=O)(=O)N)/C(=C/CNC(=O)c1ccccc1)/C(=O)N2	-10.28	29.15	0.10	0.01	490.54	9	5
Oxind_20_1	c12c(cc(cc1)C(=O)NCCc1ccc(cc1)N)/C(=C/Cc1cc(ccc1)C(=O)O)/C(=O)N2	-10.4	23.81	1.26	0.39	441.49	7	5
Oxind_20_2	c12c(cc(cc1)C(=O)NCCc1ccc(cc1)N)/C(=C/CCCc1nc[nH]c1)/C(=O)N2	-9.96	50.03	2.50	0.53	415.5	7	5
Oxind_20_5	c12c(cc(cc1)C(=O)NCCc1ccc(cc1)N)/C(=C/CC1=CC(=NC1=O)NC)/C(=O)N2	-10.5	20.11	0.23	0.16	429.48	8	5
Oxind_20_9	c12c(cc(cc1)C(=O)NCCc1ccc(cc1)N)/C(=C/Cc1cc(cc(c1)NC)C(F)(F)F)/C(=O)N2	-10.9	10.24	5.41	0.50	494.52	6	5
Oxind_20_10	c12c(cc(cc1)C(=O)NCCc1ccc(cc1)N)/C(=C/Cc1cc(ccc1)C(=O)O)/C(=O)N2	-10.6	16.99	3.61	0.39	441.49	7	5
Oxind_20_11	c12c(cc(cc1)C(=O)NCCc1ccc(cc1)N)/C(=C/Cc1ccnc(n1)N)/C(=O)N2	-10.3	28.18	1.81	0.46	414.47	8	6
Oxind_20_12	c12c(cc(cc1)C(=O)NCCc1ccc(cc1)N)/C(=C/CNc1ccc(cc1)Br)/C(=O)N2	-10.5	20.11	4.53	0.54	491.39	6	5
Oxind_20_13	c12c(cc(cc1)C(=O)NCCc1ccc(cc1)N)/C(=C/CC1CCNCC1)/C(=O)N2	-10.54	18.80	3.15	0.40	404.51	6	5
Oxind_20_14	c12c(cc(cc1)C(=O)NCCc1ccc(cc1)N)/C(=C/CC1CCN(CC1)S(=O)(=O)C)/C(=O)N2	-10.3	28.18	1.92	0.02	482.61	8	4
Oxind_20_15	c12c(cc(cc1)C(=O)NCCc1ccc(cc1)N)/C(=C/CCc1ccc(cc1)S(=O)(=O)N)/C(=O)N2	-10.9	10.24	2.18	0.19	490.58	8	6
Oxind_20_18	c12c(cc(cc1)C(=O)NCCc1ccc(cc1)N)/C(=C/CN(C(=O)Nc1ccccc1)C)/C(=O)N2	-10.88	10.59	3.21	0.06	469.55	8	5
Oxind_20_19	c12c(cc(cc1)C(=O)NCCc1ccc(cc1)N)/C(=C/CNC(=O)c1ccccc1)/C(=O)N2	-10.1	39.50	2.49	0.10	440.5	7	5
